# Combined effect of low-penetrant SNPs on breast cancer risk

**DOI:** 10.1038/bjc.2011.461

**Published:** 2011-11-01

**Authors:** S Harlid, M I L Ivarsson, S Butt, E Grzybowska, J E Eyfjörd, P Lenner, A Försti, K Hemminki, J Manjer, J Dillner, J Carlson

**Affiliations:** 1 Departments of Medical Microbiology and Clinical Chemistry, Lund University, SUS entrance 78, Malmö S-205 02, Sweden; 2 Region Skånes Biobank, Wallenberg Laboratory, Malmö S-205 02, Sweden; 3 Department of Surgery, Skåne University Hospital Malmö, Lund University, Malmö S-205 02, Sweden; 4 Center for Translational Research and Molecular Biology of Cancer, Maria Sklodowska-Curie Institute, Gliwice 44 101, Poland; 5 Cancer Research Laboratory, Faculty of Medicine, University of Iceland, Reykjavik 101, Iceland; 6 Department of Oncology, Norrlands University Hospital, Umeå S-901 85, Sweden; 7 Division of Molecular Genetic Epidemiology, German Cancer Research Center (DKFZ), Heidelberg 69 120, Germany; 8 Center for Primary Care Research, Lund University, Malmö S-205 02, Sweden; 9 Department of Plastic Surgery, Skåne University Hospital Malmö, Lund University, Malmö S-205 02, Sweden; 10 Department of Medical Microbiology, Lund University, Malmö S-205 02, Sweden; 11 Departments of Laboratory Medicine, Medical Epidemiology and Biostatistics, Karolinska Institutet and Karolinska Hospital, Stockholm S-141 86, Sweden; 12 Labmedicin Skåne, Clinical Chemistry in Lund, Lund University, Lund S-221 85, Sweden

**Keywords:** breast cancer, hereditability, common variants, multiple risk-allele GWA replication

## Abstract

**Background:**

Although many low-penetrant genetic risk factors for breast cancer have been discovered, knowledge about the effect of multiple risk alleles is limited, especially in women <50 years. We therefore investigated the association between multiple risk alleles and breast cancer risk as well as individual effects according to age-approximated pre- and post-menopausal status.

**Methods:**

Ten previously described breast cancer-associated single-nucleotide polymorphisms (SNPs) were analysed in a joint European biobank-based study comprising 3584 breast cancer cases and 5063 cancer-free controls. Genotyping was performed using MALDI-TOF mass spectrometry, and odds ratios were estimated using logistic regression.

**Results:**

Significant associations with breast cancer were confirmed for 7 of the 10 SNPs. Analysis of the joint effect of the original 10 as well as the statistically significant 7 SNPs (rs2981582, rs3803662, rs889312, rs13387042, rs13281615, rs3817198 and rs981782) found a highly significant trend for increasing breast cancer risk with increasing number of risk alleles (*P*-trend 5.6 × 10^−20^ and 1.5 × 10^−25^, respectively). Odds ratio for breast cancer of 1.84 (95% confidence interval (CI): 1.59–2.14; 10 SNPs) and 2.12 (95% CI: 1.80–2.50; 7 SNPs) was seen for the maximum *vs* the minimum number of risk alleles. Additionally, one of the examined SNPs (rs981782 in HCN1) had a protective effect that was significantly stronger in premenopausal women (*P*-value: 7.9 × 10^−4^).

**Conclusion:**

The strongly increasing risk seen when combining many low-penetrant risk alleles supports the polygenic inheritance model of breast cancer.

In addition to the highly penetrant (BRCA1, BRCA2 and TP53) and moderately penetrant (CHEK2, ATM, BRIP1 and PALB2) genetic variants conferring increased risk for breast cancer, low-penetrant risk has been linked with common genetic variants (e.g., FGFR2, TOX3, MAP3K1 and LSP1) by genome-wide association studies (GWASs) (Easton *et al*, 2007; Stacey *et al*, 2007; Turnbull and Rahman, 2008; Ghoussaini and Pharoah, 2009).

Early GWASs on breast cancer reported findings of several new breast cancer susceptibility loci (Easton *et al*, 2007; Hunter *et al*, 2007). Subsequent studies rapidly confirmed these results and added new potential risk alleles (Gold *et al*, 2008; Ahmed *et al*, 2009; Zheng *et al*, 2009; Hemminki *et al*, 2010; Long *et al*, 2010; Turnbull *et al*, 2010). Low-risk alleles in at least 25 different loci (>35 single-nucleotide polymorphisms (SNPs)) have now been identified through GWASs (Hindorff *et al*, 2011). Together, they are thought to represent roughly 8% of the familial breast cancer cases, a proportion that might increase somewhat when the true causal variants are identified (Ghoussaini and Pharoah, 2009; Turnbull *et al*, 2010). The polygenic model of inheritance, in which each variant contributes a small risk in many individuals, is often invoked to account for a substantial amount of the population attributable risk (PAR) (Dragani *et al*, 1996; Fletcher and Houlston, 2010).

The six common susceptibility loci reported in 2007 by Easton *et al* (2007), Hunter *et al* (2007) and Stacey *et al* (2007) have been verified in other studies (Gorodnova *et al*, 2010; Hemminki *et al*, 2010; Turnbull *et al*, 2010; Fletcher *et al*, 2011). The present large study, based on five well-defined study populations from Northern Europe, first aimed to investigate the significance of eight SNPs from these loci, three additional SNPs with *P*<0.05 in phase 3 of Easton *et al* (2007), and a variant in CASP8 discovered by the candidate gene approach (Cox *et al*, 2007), with special reference to age-approximated menopausal status. Furthermore, we wished to address the potential polygenic inheritance of genetic risk factors and breast cancer, that is, the association between an increasing number of risk alleles and breast cancer risk. Two studies of this issue (Reeves *et al*, 2010; Wacholder *et al*, 2010) have reported that multiple low-risk alleles do indeed increase breast cancer risk; however, neither of them included women <50 years of age. We therefore set out to perform a large investigation of the polygenic inheritance of breast cancer in women of a wide age span.

## Materials and methods

### Study populations

The study was performed within the European network of excellence Cancer Control using Population-based Registries and Biobanks (CCPRB). A total of 9395 samples (3882 cases and 5513 controls) were selected for genotyping (Table 1). The study was approved by an ethical institutional review board in each participating country and the following study populations were included.

#### MDCS

The Malmö Diet and Cancer Study (MDCS) is a prospective cohort study initiated in 1991. It comprises a total of 17 035 female residents of Malmö Sweden recruited between 1991 and 1996 (Berglund *et al*, 1993; Manjer *et al*, 2001). By linkage to the national cancer registry until 31 December 2007, 730 incident cases of invasive breast cancer were identified among MDCS participants and subsequently matched to 1460 controls from the same cohort according to sex, age (±6 months) and date of sampling at baseline (±2 months). Median age at breast cancer diagnosis was 65 years (range 45–84). In all, 33 cases and 65 controls were ⩽50 years of age at the time of diagnosis.

#### MPP

The Malmö Preventive Project (MPP) is a preventive case-finding programme started in 1974 (Berglund *et al*, 2000). Between 1977 and 1992, 10 902 women were recruited and more than 40% attended a re-examination (started in 2002) that included storing samples for DNA analysis (Nilsson *et al*, 2006; Pukkala *et al*, 2007). Among those women distinct from participants in MDCS and for whom DNA samples were available, 215 prospective invasive breast cancer cases (median age 61 years, range 32–79, 25 age ⩽50 years) were identified by cancer registry linkage up until 31 December 2007 and subsequently matched to 430 controls (50 age ⩽50 years). Matching criteria were: sex, age (±6 months) and date of sampling at baseline (±2 months). Together with the MDCS they comprise the Southern Swedish cohort.

The MDCS/MPP and the present analyses were approved by the Ethical Committee at Lund University (LU 51-90, Dnr 2009/652 and Dnr 2009/682); when donating blood, participants also signed a general consent form allowing research on their samples.

#### NSHDS

The North Sweden Health and Disease Study (NSHDS) include the Västerbotten Intervention Programme (VIP) and the Mammography Screening Programme (MSP), initiated in 1985 and 1995, respectively. Participants in the VIP are screened at 40, 50 and 60 years of age and mammography screening and blood sampling is performed among women between 50 and 69 years of age (Pukkala *et al*, 2007). Through linkage with the cancer registry up to 31 December 2008, 1680 prospective cases of invasive breast cancer (median age 56 years, range 27–95) were identified and subsequently matched to 2369 controls by sex, age (±6 months) and date of sampling at baseline (±2 months; 474 cases and 606 controls ⩽50 years of age. The NSHDS and the present analyses were approved by the Ethical Committee at Umeå University (Dnr 2010-147-132 and 07-141); when donating blood, participants also signed a general consent form allowing research on their samples.

#### ICELAND

The Icelandic samples were collected between 1998 and 2006 and represents 45–77% of all Icelandic women with invasive breast cancer diagnosed between 1957 and 2007. The rate of participation varied somewhat depending on the year of diagnosis and was highest between 1999 and 2003 (77%). Unmatched controls were collected between 2000 and 2004, either from women who participated in the population-based cervical or breast cancer screening programme and found free of breast cancer or from older women in retirement homes who had not been diagnosed with breast cancer, to generally reflect the ages of the cases. By linkage to the Icelandic cancer registry in 2008, we identified cases diagnosed before 31 December 2007. A total of 866 cases (median age 55 years, range 22–98, 314 ⩽50 years) and 948 controls (median age 58 years, range 25–102, 256 ⩽50 years) had DNA available and were eligible to us.

The use of these samples was approved by the data protection (200605037) and Science Ethics Committee in Reykjavik (VSNb2006050001/03-16 and VSNb2005070008/03-16).

#### POLAND

Cases with early onset or familial breast cancer, free from BRCA1/2 mutations, were recruited at the genetic counselling clinic in Silesia between 1997 and 2006. This collection included 391 cases (median age 46 years, range 22–81, 315 ⩽50 years) that were used in the present study. Samples from 306 unmatched controls (median age 43 years, range 18–71, 233 ⩽50 years) were collected between 2003 and 2009 from healthy women attending the same clinic, but who had no family history of breast cancer.

The use of the Polish samples was approved by the Bioethical Commission at the Centre of Oncology in Gliwice (20 November 2001). All subjects signed an informed consent form before donating their samples.

### SNP selection

All GWAS-identified loci associated with breast cancer and published before 31 June 2007 were initially included in the study (Easton *et al*, 2007; Hunter *et al*, 2007; Stacey *et al*, 2007). Individual SNPs were selected from the publications by Easton *et al* (2007) and Stacey *et al* (2007). This primary selection included 11 GWAS-identified SNPs. Three of these (rs3803663, rs12443621 and rs8051542), all situated in TOX3, have been shown to exhibit linkage (Easton *et al*, 2007; Reeves *et al*, 2007), and rs12443621 and rs80515442 were consequently excluded from further analysis. One SNP in CASP8 identified using the candidate gene approach was also included (Cox *et al*, 2007). The final selection therefore consisted of 10 SNPs (Table 2).

### Assay design and genotyping

The SEQUENOM MassARRAY Designer software (San Diego, CA, USA) included eight of the above SNPs in a single multiplex assay. The SNP analyses were performed on a MALDI-TOF mass spectrometer (SEQUENOM MassArray) using standard iPLEX reagents and protocol (SEQUENOM) and 10 ng DNA as PCR template. Primer sets were from Metabion (Martinsried, Germany).

The SNPs rs2981582 and rs1045485 were analysed by a separate TaqMan ‘assay by design’ genotyping assay on a 7900HT instrument, using Master Mix No UNG from Applied Biosystems (Foster City, CA, USA) according to the manufacturer's instructions. Reaction mixtures (6 *μ*l) containing 2 ng of DNA template and primers (rs2981582 forward primer 5′-CAGCACTCATCGCCACTTAATG-3′, reverse primer 5′-GACACCACTCGGACTGCT-3′, and probes 5′-VIC-TCTCCGCAAACAGG-MGB-3′ and 5′-FAM-CTCTCCACAAACAGG-MGB-3′) (rs1045485 forward primer 5′-ACCACGACCTTTGAAGAGCTT-3′, reverse primer 5′-ACTGTGGTCCATGAGTTGGTAGAT-3′, and probes 5′-VIC-CCCCACGATGACTG-MGB-3′ and 5′-FAM-CCCCACCATGACTG-MGB-3′) were subjected to 2 min at 50 °C and 10 min at 95 °C, followed by 50 PCR cycles of 95 °C for 15 s and 60 °C for 1 min.

### Quality control

Approximately 3% of samples from the NSHDS, 5% of the samples from Iceland and 8% of the Polish cases (total *N*=270) were included as blinded duplicates to assess the quality of the genotyping assay.

### Statistical analysis

Individual samples producing results in <80% of the assays were excluded before statistical analyses in order to eliminate samples with poor-quality DNA and in concordance with Easton *et al* (2007). Genotype data from control samples were tested for consistency with Hardy–Weinberg equilibrium (HWE) using a *χ*^2^ *P*-value cutoff of 0.001. Unconditional logistic regression models were used to measure the association between genotype for each SNP and the risk for breast cancer, using homozygotes for the common allele as reference, with adjustments for age and cohort. The material was stratified for age, ⩽50 *vs* >50 years, as a proxy for menopausal status. Furthermore, the analyses were repeated separately in each cohort. Per allele odds ratio (OR) and *P*-trend was calculated using 0, 1 or 2 copies of the minor allele as a continuous variable. The OR of <1.0 indicates that the major allele is the risk allele. To examine heterogeneity between the age groups, adjusted case–case models using unconditional logistic regression analysis were used and *P*-values of <0.05 were considered statistically significant. The *P*-value for heterogeneity (*P*_het_) of OR between cohorts was calculated using the Breslow–Day test.

For each participant the total number of risk alleles was calculated, and logistic regression was used to estimate OR and *P*-trend for each numerical group of risk alleles. The same calculation was also performed using only the seven SNPs exhibiting significance. The maximum number of risk alleles was 20 and 14, respectively, that is, 2 for each SNP. Breast cancer risk for individuals with up to ⩾11/8 risk alleles was compared with the group with ⩽6/3 risk alleles. The median number of risk alleles among both cases and control population was 8 (model including all 10 SNPs) and 5 (model including 7 SNPs), and in order to estimate the risk increase/decrease in individuals with the highest and lowest numbers of risk alleles, 8/5 risk alleles was also set as a reference. The women were also stratified according to age (⩽50 *vs* >50 years) to assess potential differences in penetrance between age groups with increasing numbers of risk alleles.

To compare estimated risks in the present study with previous reports, OR and *P*-values for trends reported in original reports are presented together with the results of the present analyses.

## Results

Of the initial 9395 samples selected for the project, 8647 (92.0%) were successfully retrieved and genotyped for ⩾80% of the SNPs. All SNPs had genotyping success rates of >90%, with an average of 97.8%. Results of all 3240 analyses performed on the 270 duplicate samples were in 100% concordance. All SNPs but one (rs4666451) passed the HWE cutoff (*P*<0.001).

Associations between seven of the reported SNPs and breast cancer were replicated in our material, with age-adjusted ORs for these SNPs in close proximity to ORs previously described (Cox *et al*, 2007; Easton *et al*, 2007; Stacey *et al*, 2007). The *P*-trend value for four of the SNPs (rs2981582, rs3803662, rs889312 and, rs13281615) was <0.001 and for the remaining three SNPs (rs13387042, rs3817198 and rs981782) was <0.01 (Table 2).

One of the SNPs (rs30099) exhibited an age-adjusted OR near to what was originally reported (Easton *et al*, 2007), but it did not pass the significance threshold of 0.05 (Table 2).

Associations of the two remaining SNPs with breast cancer were not replicated. The SNP rs1045485 (CASP8) did not reach significance, although the point estimate of the per-allele OR among women >50 years (0.92, 95% CI: 0.82–1.02) approaches that initially described by Cox *et al* (2007) (0.88, 95% CI: 0.84–0.92). Minor allele frequency (MAF) in our material was 0.24. The final SNP (rs4666451) had 5.8% missing values, failed the HWE cutoff (*P*<0.001) and had an OR that deviated from that reported (MAF was 0.35).

### Stratification analysis

Stratification of participants into age groups ⩽50 *vs* >50 years to approximate menopausal discrimination revealed different association in young *vs* older women for one of the SNPs (rs981782), whose protective effect was more pronounced in younger (per allele OR 0.82, 95% CI: 0.73–0.93) than in older women (homozygous OR 0.94, 95% CI: 0.87–1.01; Table 3). The difference was statistically significant with a *P*-value of 7.9 × 10^−4^.

**Figure 1:**
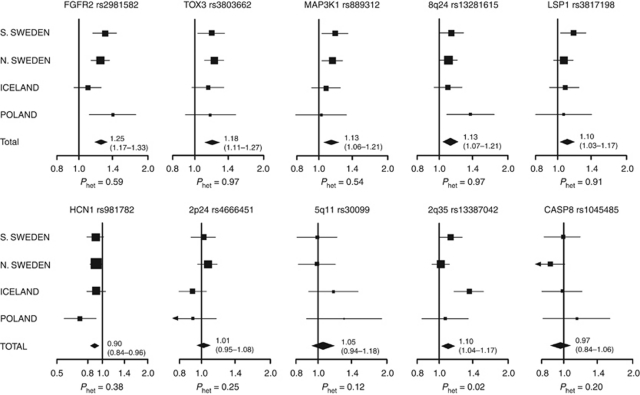
Per allele OR and 95% CI for all SNPs by participating cohorts. The area of the square for each study-population is proportional to the inverse of the variance of the estimate. Horizontal lines represent 95% CI and diamonds represent the summary OR.

Stratification of results according to study population (Figure 1) revealed similar effects for most SNPs, although rs13387042 was most strongly associated with risk in the Icelandic samples (*P*_het_=0.02). The original data set was also adjusted for study population but no difference in results was seen compared with the age-adjusted or unadjusted analysis (results not shown).

**Figure 2:**
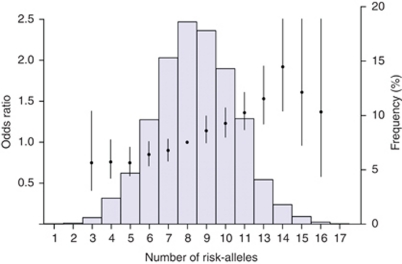
The distribution of risk alleles from the 10 SNPs amongst all women analysed in our study populations (*n*=8647), as well as the OR associated with having a certain number of risk alleles compared with the median number (8). Odds ratios are depicted by filled circles and 95% confidence intervals by black lines.

Finally, both cases and controls were classified according to the individual burden of risk alleles including both all 10 original SNPs and the SNPs statistically significantly associated with risk within this study (rs2981582, rs3803662, rs889312, rs13387042, rs13281615, rs3817198 and rs981782). A successive increase in point estimate from an OR of 1 for the group with the minimum number of risk alleles (⩽6/3 alleles) to an OR of 1.84 (95% CI 1.59–2.14; 10 SNP analysis) and 2.12 (95% CI: 1.80–2.50; 7 SNP analysis) for the group carrying the maximum number of risk alleles (⩾11/8 risk alleles) was detected (overall *P* for trend: 5.6 × 10^−20^ and 1.5 × 10^25^, respectively; Table 3a and b). When the mean number of risk alleles in the population was used as the reference (in the model including the significant seven SNPs), the maximum risk increase was 1.42 (95% CI: 1.22–1.66) for ⩾3 risk alleles above mean and a maximum protection of 0.67 (0.58–0.78) for women with ⩾2 risk alleles below mean. Results from the 10 SNP analyses were highly similar (Table 3a). The overall frequency distribution of odds ratios in the 10 SNP model is shown in Figure 2. We found no significant difference between age groups when the women were stratified according to age (⩽50 *vs* >50 years; results not shown).

## Discussion

Our study replicated the breast cancer association of 7 out of 10 previously described low risk alleles (Cox *et al*, 2007; Easton *et al*, 2007; Stacey *et al*, 2007), with nearly identical point estimates as the original studies. By comparing the total number of risk alleles in cases and controls, a highly significant increasing risk for breast cancer with an increasing number of risk alleles was seen. Calculations were primarily based on the original set of 10 SNPs and the observed association is compatible with a polygenic contribution to breast cancer in the absence of highly penetrant cancer genes (Dragani *et al*, 1996; Turnbull and Rahman, 2008; Ghoussaini and Pharoah, 2009). We also performed risk-score calculations using only the seven SNPs that originally reached significance in our study and the results were an even stronger risk trend, indicating that it might be useful to construct selective SNP panels for different populations. In this discussion, ORs are compared with the group with lowest number of risk alleles as the study population is enriched for breast cancer compared with a total background population.

The intergenic SNP rs981782 in HCN1 on 5p12, a region previously yielding significant SNPs for breast and other cancers (Ghoussaini and Pharoah, 2009), was one of the three SNPs we studied that had secondary significance in the study of Easton *et al* (2007). We found that the protective effect of the minor allele was notably more pronounced in premenopausal breast cancer (women ⩽50 years), despite the fact that this group included only 2232 individuals compared with 6398 individuals in the age group of >50 years. The *P*-value (7.9 × 10^−4^) for heterogeneity between age groups was highly significant. Previous reports did not find this difference, which could be because of differences in age stratification and/or inclusion (Easton *et al*, 2007; Reeves *et al*, 2010; Wacholder *et al*, 2010). In a fine mapping of the region, Stacey *et al* (2008) identified two SNPs in the same region (rs4415084 and rs10941679) as possible causal variants behind this association, and linked these SNPs to higher risk of ER-receptor-positive breast cancer.

SNP rs13387042 on 2q35, originally reported by Stacey *et al* (2007), was identified in a screening panel containing 1600 Icelandic women and verified in a large panel of 4554 cases and 17 577 controls containing Icelandic as well as non-Icelandic women. Our results for the Swedish and Polish cohorts differed from the Icelandic population (*P*_het_=0.02), whose carriers of the rs13387042 A allele demonstrate an increased risk. The 2q35 locus has also been verified in other non-Icelandic populations (Milne *et al*, 2009), indicating that this SNP is generally associated with breast cancer. Nevertheless, the significantly higher risk that we found in Iceland is noteworthy.

For SNP rs1045485 in CASP8, originally discovered by Cox *et al* (2007) through candidate gene analysis, we found a similar point estimate as in the original study for women >50 years of age, although the association with breast cancer did not achieve significance in our cohorts. A recent meta-analysis (Sergentanis and Economopoulos, 2009) concluded that CASP8 rs1045485 does reduce the risk of breast cancer in minor allele carriers, at least in Caucasian populations.

Our study includes cases and controls from five different study populations in three different countries, representing different northern European inhabitants. Each cohort has its own strengths and weaknesses. The Swedish NHSDS and MDCS cohorts have matched controls to cases in the same prospective population-based study, age and duration of follow-up. Enrolment in the MDCS has shown a slight selection towards higher socioeconomic status than the general population, but this selection is the same for cases and controls (Manjer *et al*, 2001). The MDCS participants were recruited at age 44–65 years. The exclusion of prevalent cases removes early breast cancer cases from this population. Although the NHSDS participants were primarily included from age 40 and upwards, the mammography subcohort included some case as young as 27 years. In Iceland, prevalent cases of breast cancer were recruited at varying times after diagnosis, resulting in an exclusion of early lethal cases and older women with other causes of death. A similar bias is present in the MPP cohort despite prospective population-based design, as DNA samples were acquired from only ∼40% of cases and matched controls participating in a follow-up visit. It is therefore possible that these two study populations are biased towards breast cancer cases with more favourable outcome. The Polish cases are recruited from families with multiple breast cancer cases, or because of early onset of breast cancer, something that seems to strengthen the association between rs981782 and breast cancer in women ⩽50 years that is especially prominent in this cohort (Figure 1).

Methodological strengths include the exclusion of samples with <80% successful genotypes and by 100% concordant genotypes in 270 duplicate samples. Although the use of *P*<0.05 as significance limit is appropriate for a replication study verifying reported associations, the occurrence of false negative findings cannot be excluded. Lack of significance, in particular of the CASP8 (rs1045485) association, might be attributable to insufficient statistical power.

The FGFR2 and TOX3 SNPs have consistently been verified in published reports (Huijts *et al*, 2007; Gorodnova *et al*, 2010; Hemminki *et al*, 2010; Turnbull *et al*, 2010), whereas replication of the other low-penetrant SNPs has been less constant. At least two previous studies (Reeves *et al*, 2010; Wacholder *et al*, 2010) have analysed the association between the number of risk alleles and overall breast cancer risk. Wacholder *et al* (2010) analysed almost 6000 women with breast cancer aged 50–79 years. They had highly similar results to ours, but pointed out the fact that addition of a risk score obtained from adding genotypes from 10 low-penetrant SNPs contributed little to breast cancer risk prediction over and above the established clinical risk prediction models that include age at first childbirth, Gail score and the number of first-degree relatives with breast cancer.

In the present study we used a simple addition of the number of risk alleles, but still obtained almost exactly the same result as the study of Reeves *et al* (2010), although these authors took into account the magnitudes of the individual SNP effects. Thus, the additive approach appears to be sufficient for risk score calculation.

Our findings, including total risk score, are well in line with previous studies. A novel finding for this study was the fact that the protective effect of the HCN1 SNP rs981782 was significantly stronger in women ⩽50 years of age. Odds ratios presented both here and in the other two studies consistently show that total risk scores based on low-penetrant SNPs adds only very modest improvement to risk prediction models based on medical data, and are therefore not likely to have an immediate clinical use. However, we can show that simple calculation of the number of risk alleles gives highly reproducible risk scores between studies and could be useful in further studies of the genetic predisposition to breast cancer.

## Figures and Tables

**Table 1:** Characteristics of participating cohorts

**Cohort**	**Country**	**Region**	**Date of last follow-up**	**Number of cases**	**Number of controls**	**Age (cases) median (range)**	**Age (controls) median (range)**
MDCS	Sweden	Southern	31 December 2007	730	1460	63 (45–84)	63 (45–84)
MPP	Sweden	Southern	31 December 2007	215	430	61 (32–79)	61 (32–79)
NSHDS	Sweden	Northern	31 December 2008	1680	2369	56 (27–95)	58 (27–83)
ICELAND	Iceland	Whole country	31 December 2007	866	948	55 (25–93)	58 (22–98)
POLAND	Poland	South Western	31 December 2004	391	306	46 (22–81)	43 (18–71)
Total^a^	—	—	—	3882	5513	57 (22–95)	60 (18–98)

Abbreviations: MDCS=Malmö Diet and Cancer Study; MPP=Malmö Preventive Project; NSHDS=North Sweden Health and Disease Study.

a Includes samples later omitted because of poor DNA quality.

**Table 2:** Odds ratio and 95% CI for breast cancer for all SNPs

				**Reference samples**		**CCPRB samples**							
**Reference**	**Gene/region** **rs no.**	**Genotype**		**OR (95% CI)**	***P*-trend**	***n* (cases/controls)** **MV (total %)**	**OR (95% CI)** **All age adjusted**	***P*-trend**	**OR (95% CI)** ⩽**50 years^a^**	***P*-trend**	**OR (95% CI)** **>50 years^b^**	***P*-trend**	***P*_het_** ⩽**50 *vs* >50**
Easton *et al* (2007)	FGFR2	Ref allele	AA	1		1058/1820	1		1		1		
	2981582	Het	Aa	1.23 (1.18–1.28)		1777/2404	1.27 (1.15–1.40)		1.51 (1.25–1.83)		1.19 (1.06–1.34)		
		Homo	aa	1.63 (1.53–1.72)		733/809	1.54 (1.36–1.75)		1.60 (1.25.-2.03)		1.53 (1.32–1.77)		
		Per allele		1.26 (1.23–1.30)	2.0 × 10^−76^	MV: 16/30 (0.5%)	1.25 (1.17–1.33)	2.9 × 10^−12^	1.29 (1.15–1.46)	2.4 × 10^−5^	1.23 (1.14–1.32)	1.8 × 10^−8^	0.16
Easton *et al* (2007)	TOX3	Ref allele	AA	1		1794/2768	1		1		1		
	3803662	Het	Aa	1.23 (1.18–1.29)		1420/1898	1.15 (1.05–1.26)		1.10 (0.92–1.31)		1.17 (1.06–1.31)		
		Homo	aa	1.39 (1.26–1.45)		330/352	1.46 (1.24–1.72)		1.77 (1.28–2.45)		1.37 (1.14–1.66)		
		Per allele		1.20 (1.16–1.24)	1.0 × 10^−36^	MV: 40/45 (1.0%)	1.18 (1.11–1.27)	9.6 × 10^−7^	1.22 (1.07–1.39)	3.1 × 10^−3^	1.17 (1.08–1.27)	7.4 × 10^−5^	0.98
Easton *et al* (2007)	MAP3K1	Ref allele	AA	1		1767/2687	1		1		1		
	889312	Het	Aa	1.13 (1.09–1.18)		1478/1938	1.18 (1.07–1.29)		1.14 (0.95–1.36)		1.19 (1.07–1.32)		
		Homo	aa	1.27 (1.19–1.36)		309/390	1.21 (1.03–1.42)		1.41 (1.02–1.94)		1.15 (0.96–1.39)		
		Per allele		1.13 (1.10–1.16)	7.0 × 10^−20^	MV: 30/48 (0.9%)	1.13 (1.06–1.21)	3.2 × 10^−4^	1.16 (1.02–1.33)	0.02	1.12 (1.04–1.21)	4.7 × 10^−3^	0.12
Easton *et al* (2007)	8q24	Ref allele	AA	1		1103/1766	1		1		1		
	13281615	Het	Aa	1.06 (1.01–1.11)		1723/2357	1.15 (1.05–1.27)		1.05 (0.86–1.27)		1.20 (1.07–1.35)		
		Homo	aa	1.18 (1.10–1.25)		719/884	1.28 (1.13–1.45)		1.28 (1.01–1.62)		1.28 (1.10–1.48)		
		Per allele		1.08 (1.05–1.11)	5.0 × 10^−12^	MV: 39/56 (1.1%)	1.13 (1.07–1.21)	5.6 × 10^−5^	1.12 (1.00–1.26)	0.05	1.14 (1.06–1.22)	3.7 × 10^−4^	0.05
Easton *et al* (2007)	LSP1	Ref allele	AA	1		1622/2418	1		1		1		
	3817198	Het	Aa	1.06 (1.02–1.11)		1505/1986	1.13 (1.03–1.24)		1.06 (0.89–1.28)		1.15 (1.03–1.28)		
		Homo	aa	1.17 (1.08–1.25)		357/458	1.16 (1.00–1.35)		1.28 (0.96–1.72)		1.10 (0.92–1.32)		
		Per allele		1.07 (1.04–1.11)	3.0 × 10^−9^	MV: 100/201 (3.5%)	1.10 (1.03–1.17)	6.2 × 10^−3^	1.11 (0.97–1.26)	0.12	1.09 (1.01–1.17)	0.04	0.19
Easton *et al* (2007)	HCN1	Ref allele	AA	1		960/1256	1		1		1		
	981782	Het	Aa	0.96 (0.92–1.01)		1795/2491	0.95 (0.85–1.05)		0.88 (0.72–1.07)		0.98 (0.87–1.11)		
		Homo	aa	0.92 (0.87–0.97)		691/1132	0.80 (0.71–0.91)		0.67 (0.52–0.85)		0.87 (0.75–1.01)		
		Per allele		0.96 (0.93–0.99)	9.0 × 10^−6^	MV: 138/184 (3.7%)	0.90 (0.84–0.96)	1.0 × 10^−3^	0.82 (0.73–0.93)	1.9 × 10^−3^	0.94 (0.87–1.01)	0.08	7.9 × 10^−3^
Easton *et al* (2007)	2p24	Ref allele	AA	1		1381/1980	1		1		1		
	4666451	Het	Aa	0.98 (0.93–1.02)		1485/2122	1.01 (0.91–1.11)		0.99 (0.82–1.19)		1.01 (0.91–1.13)		
		Homo	aa	0.93 (0.87–0.99)		489/686	1.02 (0.89–1.17)		1.11 (0.85–1.44)		1.00 (0.85–1.17)		
		Per allele		0.97 (0.94–1.00)	6.0 × 10^−5^	MV: 229/275 (5.8%)	1.01 (0.95–1.08)	0.75	1.04 (0.92–1.17)	0.57	1.00 (0.93–1.08)	0.95	0.93
Easton *et al* (2007)	5q11	Ref allele	AA	1		3023/4302	1		1		1		
	30099	Het	Aa	1.06 (1.00–1.11)		524/706	1.06 (0.94–1.20)		1.26 (0.99–1.61)		1.00 (0.87–1.16)		
		Homo	aa	1.09 (0.96–1.24)		28/38	1.03 (0.63–1.68)		1.47 (0.59–3.68)		0.89 (0.49–1.62)		
		Per allele		1.05 (1.01–1.10)	1.0 × 10^−3^	MV: 9/17 (0.3%)	1.05 (0.94–1.18)	0.35	1.25 (1.01–1.56)	0.04	0.99 (0.87–1.13)	0.88	0.38
Stacey *et al* (2007)	2q35	Ref allele	AA	1		796/1230	1		1		1		
	13387042	Het	Aa	1.11 (1.03–1.20)		1590/2328	1.05 (0.94–1.17)		1.00 (0.81–1.23)		1.07 (0.94–1.22)		
		Homo	aa	1.44 (1.30–1.58)		1007/1279	1.21 (1.07–1.37)		1.12 (0.88–1.41)		1.25 (1.08–1.44)		
		Per allele		1.20 (1.14–1.26)	4.5 × 10^−14^	MV: 191/226 (4.8%)	1.10 (1.04–1.17)	1.9 × 10^−3^	1.06 (0.94–1.19)	0.34	1.12 (1.04–1.20)	2.3 × 10^−3^	0.57
Cox *et al* (2007)	CASP8	Ref allele	AA	1		2752/3836	1		1		1		
	1045485	Het	Aa	0.89 (0.85–0.94)		759/1093	0.97 (0.87–1.07)		1.17 (0.96–1.44)		0.90 (0.80–1.02)		
		Homo	aa	0.74 (0.62–0.87)		46/70	0.93 (0.63–1.35)		0.98 (0.45–2.17)		0.93 (0.60–1.43)		
		Per allele		0.88 (0.84–0.92)	1.1 × 10^−7^	MV: 27/64 (1.1%)	0.97 (0.88–1.06)	0.46	1.14 (0.94–1.37)	0.18	0.92 (0.82–1.02)	0.12	0.38

Abbreviations: CCPRB=Cancer Control using Population-based Registries and Biobanks; CI=confidence interval; Het=heterozygote; Homo=homozygote; MV=missing value; OR=odds ratio; *P*_het_=*P*-value for heterogeneity; Ref=reference; SNP=single-nucleotide polymorphism.

a Total of 2232 samples.

b Total of 6398 samples.

**Table 3:** Number of risk alleles and breast cancer risk for (a) 10^a^ SNP analysis and (b) 7^b^ SNP analysis

	**Cases**		**Controls**		**OR (95% CI)**	**OR (95% CI)**
**No. of risk alleles**	***N***	**%**	***N***	**%**	**Breast cancer**	**Breast cancer**
**(a)**						
					Reference=8 risk alleles^c^	Reference ⩽6 risk alleles
⩽6	521	14.5	983	19.4	0.80 (0.69–0.92)	1
7	494	13.8	831	16.4	0.90 (0.77–1.04)	1.12 (0.96–1.31)
8	641	17.9	966	19.1	1	1.25 (1.08–1.45)
9	662	18.5	873	17.2	1.14 (0.99–1.32)	1.43 (1.24–1.66)
10	555	15.5	682	13.5	1.23 (1.06–1.42)	1.54 (1.32–1.79)
⩾11	711	19.8	728	14.4	1.47 (1.27–1.70)	1.84 (1.59–2.14)
						*P*-trend: 5.6 × 10^−20^
**(b)**						
					Reference=5 risk alleles^c^	Reference ⩽3 risk alleles
⩽3	438	12.2	916	18.1	0.67 (0.58–0.78)	1
4	539	15.0	929	18.3	0.81 (0.71–0.94)	1.21 (1.04–1.42)
5	759	21.2	1065	21.0	1	1.49 (1.29–1.73)
6	723	20.2	963	19.0	1.05 (0.92–1.20)	1.57 (1.35–1.82)
7	586	16.4	659	13.0	1.25 (1.08–1.44)	1.86 (1.59–2.18)
⩾8	539	15.0	531	10.5	1.42 (1.22–1.66)	2.12 (1.80–2.50)
						*P*-trend: 1.5 × 10^−25^

Abbreviations: CI=confidence interval; OR=odds ratio; SNP=single-nucleotide polymorphism.

a rs2981582, rs3803662, rs889312, rs13281615, rs3817198, rs981782, rs13387042, rs4666452, rs30099 and rs1045485.

b rs2981582, rs3803662, rs889312, rs13281615, rs3817198, rs981782 and rs13387042

c Median number of risk alleles.
